# Severity of malocclusion in adolescents: populational-based study in the north of Minas Gerais, Brazil

**DOI:** 10.1590/S1518-8787.2016050005861

**Published:** 2016-04-15

**Authors:** Marise Fagundes Silveira, Rafael Silveira Freire, Marcela Oliveira Nepomuceno, Andrea Maria Eleutério de Barros Lima Martins, Luiz Francisco Marcopito

**Affiliations:** I Departamento de Ciências Exatas. Universidade Estadual de Montes Claros. Montes Claros, MG, Brasil; II Pós-Graduação em Ciências da Saúde. Universidade Estadual de Montes Claros. Montes Claros, MG, Brasil; III Programa de Iniciação Científica em Medicina. Faculdades Integradas Pitágoras de Montes Claros. Montes Claros, MG, Brasil; IV Departamento de Odontologia. Universidade Estadual de Montes Claros. Montes Claros, MG, Brasil; V Departamento de Medicina Preventiva. Escola Paulista de Medicina. Universidade Federal de São Paulo. São Paulo, SP, Brasil

**Keywords:** Adolescent, Malocclusion, epidemiology, Risk Factors, Socioeconomic Factors, Self Concept, Cross-Sectional Studies

## Abstract

**OBJECTIVE:**

To identify the factors associated with severity of malocclusion in a population of adolescents.

**METHODS:**

In this cross-sectional population-based study, the sample size (n = 761) was calculated considering a prevalence of malocclusion of 50.0%, with a 95% confidence level and a 5.0% precision level. The study adopted correction for the effect of delineation (*deff* = 2), and a 20.0% increase to offset losses and refusals. Multistage probability cluster sampling was adopted. Trained and calibrated professionals performed the intraoral examinations and interviews in households. The dependent variable (severity of malocclusion) was assessed using the Dental Aesthetic Index (DAI). The independent variables were grouped into five blocks: demographic characteristics, socioeconomic condition, use of dental services, health-related behavior and oral health subjective conditions. The ordinal logistic regression model was used to identify the factors associated with severity of malocclusion.

**RESULTS:**

We interviewed and examined 736 adolescents (91.5% response rate), 69.9% of whom showed no abnormalities or slight malocclusion. Defined malocclusion was observed in 17.8% of the adolescents, being severe or very severe in 12.6%, with pressing or essential need of orthodontic treatment. The probabilities of greater severity of malocclusion were higher among adolescents who self-reported as black, indigenous, *pardo* or yellow, with lower *per capita* income, having harmful oral habits, negative perception of their appearance and perception of social relationship affected by oral health.

**CONCLUSIONS:**

Severe or very severe malocclusion was more prevalent among socially disadvantaged adolescents, with reported harmful habits and perception of compromised esthetics and social relationships. Given that malocclusion can interfere with the self-esteem of adolescents, it is essential to improve public policy for the inclusion of orthodontic treatment among health care provided to this segment of the population, particularly among those of lower socioeconomic status.

## RESUMO

**OBJETIVO:**

Identificar os fatores associados à gravidade da maloclusão em uma população de adolescentes.

**MÉTODOS:**

Neste estudo transversal de base populacional, o tamanho da amostra (n = 761) foi calculado considerando prevalência de maloclusão de 50,0%, com nível de 95% de confiança e precisão de 5,0%. Foi adotada correção para o efeito de delineamento (*deff* = 2), e acréscimo de 20,0% para compensar perdas e recusas. Adotou-se amostragem probabilística por conglomerado em múltiplos estágios. Profissionais treinados e calibrados realizaram os exames intrabucais e entrevistas nos domicílios. A variável dependente, gravidade da maloclusão, foi avaliada pelo Índice de Estética Dental. As variáveis independentes foram agrupadas em cinco blocos: características demográficas, condição socioeconômica, uso de serviços odontológicos, comportamento relacionado à saúde e condições subjetivas de saúde bucal. Foi utilizado modelo de regressão logística ordinal para identificar os fatores associados à gravidade da maloclusão.

**RESULTADOS:**

Foram entrevistados e examinados 763 adolescentes (taxa de resposta de 91,5%), dos quais 69,6% não apresentou anormalidades ou apresentou maloclusão leve. Maloclusão definida foi observada em 17,8% dos adolescentes, sendo grave ou muito grave em 12,6% dos adolescentes, com tratamento ortodôntico altamente necessário ou fundamental. As chances de maior gravidade de maloclusão foram maiores entre adolescentes que relataram ser negro, índio, pardo ou amarelo, ter menor renda *per capita*, ter hábitos bucais deletérios, ter percepção negativa de sua aparência e percepção do seu relacionamento social afetado pela condição bucal.

**CONCLUSÕES:**

As maloclusões graves ou muito grave foram mais prevalentes entre adolescentes com desvantagem social, que relataram hábitos deletérios, que perceberam comprometimento estético e nas relações sociais. Visto que as oclusopatias podem interferir na autoimagem dos adolescentes, torna-se fundamental o aprimoramento de políticas públicas voltadas para a inclusão do tratamento ortodôntico entre os procedimentos de saúde acessíveis a essa população, sobretudo aos menos favorecidos socioeconomicamente.

## INTRODUCTION

Although the incidence of dental caries has diminished in many countries, Brazil included, it persists as the main oral health problem among children and adolescents^9^ ^,^ ^19^. As dental health improves among the population, other oral problems may require attention, such as malocclusion^2^. Malocclusion is considered a problem related to the growth and development of maxillary or mandibular bones during childhood and adolescence^11^. This kind of anomaly may have functional, aesthetic or psychosocial effects, with negative impact on the daily life of affected individuals^16^. It is caused by an interaction of environmental, congenital, morphological, and biomechanical factors^8^.

Malocclusion can be considered a public health problem, given its high prevalence and possibilities of prevention and treatment^20^. Although the demand for orthodontic treatment in contemporary society has grown, population-based comprehensive studies investigating the prevalence of malocclusion and its association with sociodemographic factors, use of dental services and self-perceived oral health among Brazilian adolescents are still relatively scarce. Within this context, this study aimed to identify the factors associated with malocclusion in a population of Brazilian adolescents.

## METHODS

This cross-sectional population-based study used data collected in the epidemiological survey of oral health conditions among the population of Montes Claros, state of Minas Gerais, Brazil (Project SB-MOC)^14^. Montes Claros is a middle-sized town located in northern Minas Gerais, in the Sao Francisco River basin, 442 km from the state capital. It has a human development index (HDI) of 0.770 and *per capita* income of R$650.02. It is considered the region’s main economic and educational center, with economic activities focused on manufacturing, services, trade, and agriculture and livestock farming. According to the 2010 census^a^, the municipality has a resident population of 361,971 inhabitants, 34,143 of whom (9.4%) are in the 15 to 19 age group.

This epidemiological survey of oral health aimed to estimate the prevalence of various oral health problems, such as dental caries, periodontal disease, malocclusion, fluorosis, among others, in adolescents aged from 15 to 19. Sample size was calculated to estimate population parameters with a prevalence of 50.0%, 95% of confidence level and 5.0% of precision. The study adopted correction for the effect of delineation (*deff* = 2) deriving from cluster sampling, and a 20.0% increase to offset losses and refusals. A sample of at least 761 people was estimated.

A two-stage cluster sampling process was adopted. In the first stage, 52 of the 276 urban census sectors of the municipal area were chosen (sample fraction [*f*_*1*_] = 0.19) by simple random sampling (SRS). The number of sectors was defined considering the average number of households per sector and the average number of individuals per household (data based on the 2003 census and estimated for 2008). In the second stage, a sample fraction (*f*_*2*_) of the blocks in each one of the 52 chosen sectors was selected by SRS (*f*_*2*_ varied between 0.06 and 0.16), resulting in an average of seven block per sector. All the households in the selected blocks were visited in sequence and residents in the studied age group were invited to take part in the survey. In the rural area, a single-stage cluster sampling process was used. Two of the eleven identified rural areas (*f*_*1*_ = 0.18) were selected by SRS. The number of areas was defined considering the average number of households in the rural areas, the average number of individuals per household and the percentage of rural population in the municipality (data provided by the town’s epidemiological surveillance service). All households situated within 500 m of a reference institution (school) were selected and their residents aged from 15 to 19 were invited to take part in the survey. Details on the sampling procedure can be obtained in another publication^15^.

To incorporate the structure of the complex sampling plan in the data statistical analysis, each interviewee was associated to a weight *p*, corresponding to their inverse probability of inclusion in the sample (*f*). In the urban area, the selection of individuals was done in two stages. Therefore, their inclusion probability was obtained by the product of the inclusion probability in each one of the stages (*f* = *f*_1_ x *f*_2_), in which *f*_1_ = inclusion probability in the first stage and *f*_2_ = inclusion probability in the second stage. The possibility of refusal to participate was also considered, which would cause different inclusion probabilities. Thus, the response rate (*r*_response_) in each sector was incorporated and the final inclusion probability of each individual was obtained by the expression *f* = *f*_1_ x *f*_2_ x *r*_response_. In the rural area, the selection of individuals was composed of a single stage, and therefore the inclusion probability was calculated by the expression *f* = *f*_1_ x *r*_response_, considering *f*_1_ = inclusion probability in the first stage. Finally the weight of each interviewee was obtained by inverse inclusion probability (p = *1/f*)^15^.

Field work was carried out by 24 teams composed of a recorder and an examiner who were previously trained and calibrated. During training and calibration, inter- and intra-examiner agreement was estimated by intraclass correlation coefficient (ICC) for the dental aesthetic index (DAI) and the weighted kappa for their components, with an acceptable limit value of 0.60. Examiners whose agreement exceeded this limit were considered fit; the remainder were submitted to an additional calibration exercise until the acceptable limit was reached. Details of the training and calibration process can be obtained in another publication^13^.

Data were collected with a handheld computer using an especially created software program, which enabled the simultaneous and automatic construction of a database. Interviews and intraoral examinations were performed in a spacious environment with natural light with a mirror and a Community Periodontal Index (CPI) probe of the World Health Organization (WHO), previously sterilized. Data stored in the handheld computers were transferred to a main computer and then exported to PASW 17.0 software for checking, revision, and correction.

The dependent variable severity of malocclusion was evaluated according to DAI, enabling the classification of individuals into: absence of abnormality or slight malocclusion (DAI ≤ 25); defined malocclusion (DAI = 26 to 30); severe malocclusion (DAI = 31 to 35); and very severe malocclusion (DAI > 36)^13^. The dependent variables were: (a) demographic characteristics: sex (male; female), self-reported skin color (non-white; white), marital status (single; married or stable union) and age (in years); (b) socioeconomic condition: level of education (≤ 8 years of schooling; > 8 years of schooling), monthly *per capita* income (≤ R$200.00; > R$200.00), household crowding (more than one person per room; up to one person per room); (c) use of dental services: use of service (never used; used), type of dental service used (public or charity; private, health plan or insurance), time since last dentist appointment (< 1 year; ≥ 1 year), regular dentist visits (yes; no); (d) health-related behavior: tooth-brushing frequency (< 3 times/day; ≥ 3 times/day), use of dental floss (no; yes), use of topical fluoride (no; yes), harmful oral habits (yes; no), smoking habits (yes; no), alcohol consumption (yes; no), physical exercise (never or rarely; occasionally; frequently or always); and (e) oral health subjective conditions: self-perceived oral health (negative; positive), self-perceived chewing (negative; positive), self-perceived appearance of teeth or gums (negative; positive), self-perceived speech due to teeth or gums (negative; positive), self-perceived social relationship affected by oral health (affects; does not affect).

The skin color variable was classified as non-white (those who self-reported as black, *pardo*, yellow or indigenous) and white (those who self-reported as white). As to *per capita* income, the distribution median was R$200.00, a value used as the cutoff point. Self-perceived oral health, chewing, appearance and speech were considered negative when individuals rated them as very bad, bad or average; and positive when rated as good or very good.

The variables were described through their absolute and relative frequency distributions. A 95% confidence interval for the prevalence of malocclusion was also estimated. An ordinal logistic regression model (proportional odds model) was used in the analysis of factors associated with the outcome^1^. Bivariate analyses were performed and variables presenting a descriptive level below 0.20 (p < 0.20) were selected for the multiple model^7^. A stepwise forward procedure was adopted to construct the multiple regression model, i.e., the model was started with the most statistically significant variable, selected in the bivariate analysis, and the remaining variables were then added, one by one, in descending order of the descriptive level. Variables presenting significant association with the outcome (p > 0.05) were kept in the final model. Raw and adjusted odds ratios were estimated, with their respective 95% confidence intervals.

The adjustment quality of the final model was evaluated by the deviance test and the assumption of proportional odds was assessed by parallel lines tests^1^. All analyses were performed on the PASW® 17.0 statistical program, using the complex sample model to analyze data from complex samples, with the aim of adjusting the variability estimates in the cluster sampling.

This survey was approved by the ethics committee of the Universidade Estadual de Montes Claros (Opinion 318/08). All individuals participating in the survey signed an informed consent form.

## RESULTS

A total of 763 adolescents took part in the survey, 99.6% of whom lived in the urban area. The response rate was 91.5%, and the main reason for losses was failure to locate adolescents after three household visits.

The average age of adolescents was 17.1 years old, and most of them were female (52.7%), single (94.7%), self-reported non-white skin color (73.1%), attending an educational institution (73.9%), had more than eight years of schooling (77.2%) and a monthly *per capita* income equal to or below R$200.00 (58.7%). The other characteristics are described in Table 1.

**Table 1:** Distribution of adolescents aged from 15 to 19 according to demographic and socioeconomic characteristics, use of dental services, health-related behavior and oral health subjective conditions. Montes Claros, MG, Southeastern Brazil, 2009.

Variable	n^a^	%^b^
Demographic characteristic		
Sex		
Male	367	47.3
Female	396	52.7
Self-reported skin color		
Non-white	554	73.1
White	209	26.9
Marital status		
Single	729	94.7
Married or stable union	34	5.3
Socioeconomic condition		
Level of education (years of schooling)		
> 8	595	77.2
≤ 8	168	22.8
Attending school		
No	185	26.1
Yes	578	73.9
Monthly *per capita* incomec		
≤ R$200.00	374	58.7
> R$200.00	309	41.3
Household crowding		
More than one person per room	122	18.5
Up to one person per room	641	81.5
Use of dental services		
Use of dental services		
Never	46	6.1
Has used	717	93.9
Type of dental service		
Never used	46	6.1
Public or charity	366	48.0
Private, health plan or insurance	351	45.9
Time since last dentist appointment		
Never seen a dentist	46	6.1
One year or more	333	43.7
Less than one year	384	50.2
Sees dentist regularly		
No	575	75.4
Yes	188	24.6
Health-related behavior		
Tooth-brushing frequency		
2< 3 times/day	217	28.9
≥ 3 times/day	545	71.1
Use of dental floss		
No	426	58.8
Yes	336	41.2
Use of topical fluoride		
No	509	60.7
Yes	253	39.3
Harmful oral habits		
Yes	302	41.2
No	457	58.8
Smoking		
Yes	40	5.1
No	723	94.9
Alcohol consumption		
Yes	146	18.8
No	617	81.2
Physical activity		
Rarely or never	260	31.8
Occasionally	160	20.0
Always or frequently	343	48.2
Oral health subjective condition		
Self-perceived oral health		
Negative	257	34.4
Positive	505	65.6
Self-perceived chewing		
Negative	162	22.0
Positive	600	78.0
Self-perceived appearance of teeth or gums		
Negative	250	35.4
Positive	513	64.6
Self-perceived speech due to teeth or gums		
Negative	92	14.0
Positive	671	86.0
Self-perceived relationship affected by oral health		
Affects	128	17.5
Does not affect	621	82.5

^a^ Totals vary due to information losses.

^b^ Values corrected by design effect *(deff)*.

^c^ Cutoff point defined by distribution median.

Regarding severity of malocclusion, an expressive percentage (69.6%) of adolescents had no abnormalities or slight malocclusion, while defined malocclusion was observed in 17.8%. Fifty-six (6.2%) and 49 (6.4%) adolescents showed severe or very severe malocclusion, respectively. Prevalence of malocclusion conditions is presented in Table 2.

**Table 2:** Distribution of adolescents aged from 15 to 19 according to severity of malocclusion and occlusal conditions evaluated by the dental aesthetic index (DAI). Montes Claros, MG, Southeastern Brazil, 2009.

Variable	n^a^	%	IC95%^b^
Severity of malocclusion			
Absence of malocclusion or slight malocclusion	510	69.5	63.3–75.9
Defined malocclusion	134	17.8	13.4–23.0
Severe malocclusion	56	6.2	4.2–8.5
Very severe malocclusion	49	6.4	4.6–8.6
Occlusal condition			%^b^
Number of missing teeth in superior dental arch			
None	748		98.5
One or more	13		1.5
Number of missing teeth in inferior dental arch			
None	755		99.3
One or more	6		0.7
Alignment in anterior segment			
None	460		58.2
One segment	195		26.6
Two segments	106		15.2
Spacing in anterior segment			
None	618		80.8
One segment	119		16.7
Two segments	24		2.5
Midline diastema			
No	613		80.5
Yes	146		19.5
Anterior superior irregularity			
< 2 mm	567		77.4
≥ 2 mm	194		22.6
Anterior inferior irregularity			
< 2 mm	569		75.2
≥ 2 mm	192		24.8
*Maxillary* overjet			
< 4 mm	603		80.4
≥ 4 mm	158		19.6
*Mandibular* overjet			
< 4 mm	759		99.6
≥ 4 mm	3		0.4
Anterior openbite			
< 2 mm	728		95.8
≥ 2 mm	33		4.2
Molar relationship			
Normal	407		56.3
Half cusp	247		32.1
One cusp	97		11.6

^a^ Totals vary due to information losses.

^b^ Values corrected by design effect *(deff).*

Bivariate analysis results are shown in Table 3, which presents only variables in which p < 0.20, selected for the multiple analysis: skin color, level of education, *per capita* income, household crowding, time since last dentist appointment, harmful oral habits, self-perceived oral health, appearance and relationship affected by oral health.

**Table 3:** Distribution of severity of malocclusion among adolescents aged from 15 to 19 according to demographic and socioeconomic characteristics, use of dental services, health-related behavior and oral health subjective conditions. Montes Claros, MG, Southeastern Brazil, 2009.

Variable	Severity of malocclusion											

	Absent or slight		Defined		Severe		Very severe		Total	OR_r_	95%CI	p*
				
	n	%	n	%	n	%	n	%	n			
Demographic characteristics and socioeconomic condition												
Self-reported skin color												
Non-white	355	67.2	102	18.4	47	7.2	40	7.2	544	1.7	1.2–2.4	0.004
White	155	77.3	32	15.7	9	2.9	9	4.1	205	1.0	-	-
Level of education												
> 8 years of schooling	409	71.7	97	16.8	42	5.5	34	5.9	582	0.7	0.5–1.0	0.069
≤ 8 years of schooling	101	64.1	37	20.5	14	7.7	15	7.8	167	1.0	-	-
*Per capita* income												
≤ R$200.00	224	62.7	74	20.7	38	7.9	30	8.7	366	1.7	1.1–2.7	0.028
> R$200.00	218	74.0	48	15.3	18	5.5	19	5.2	303	1.0	-	-
Household crowding												
> 1 person/room	70	60.3	26	20.0	11	8.8	11	10.9	118	1.8	1.1–2.9	0.027
≤ 1 person/room	440	72.1	108	17.2	45	5.4	38	5.3	631	1.0	-	-
Use of dental services and health-related behavior												
Time since last dentist appointment												
Never seen a dentist	31	67.1	7	12.1	1	1.5	6	19.2	45	1.6	0.72–3.4	0.250
One year or more	214	67.6	57	17.0	32	8.1	24	7.4	327	1.3	1.0–1.8	0.066
Less than one year	265	72.3	70	19.0	23	4.8	19	3.9	377	1.0	-	-
Harmful oral habits												
Present	180	62.2	64	20.2	31	8.9	22	8.7	297	1.9	1.2–3.1	0.009
Absent	329	75.5	68	15.8	25	4.0	27	4.7	449	1.0	-	-
Oral health subjective condition												
Self-perceived oral health												
Negative	157	63.9	43	17.0	25	7.9	25	11.1	250	1.7	1.1–2.5	0.015
Positive	352	73.0	91	18.0	31	5.1	24	3.9	498	1.0	-	-
Self-perceived appearance of teeth or gums												
Negative	133	59.2	54	22.0	27	8.8	26	10.0	240	2.2	1.3–3.6	0.003
Positive	377	75.7	79	15.4	29	4.5	22	4.4	507	1.0	-	-
Self-perceived relationship affected by oral health												
Affects	75	58.2	26	22.2	10	6.9	17	12.7	128	2.0	1.5–2.6	0.000
Does not affect	435	72.5	108	16.7	46	5.8	32	5.0	621	1.0	-	-

OR_r_: raw odds ratio

* This table shows only variables with p ≤ 0.20 in the raw analysis.

Table 4 shows the multiple analysis results. Among the demographic and socioeconomic characteristics, we associated the following factors with greater severity of malocclusion: non-white adolescents (OR = 1.5; 95%CI 1.1–2.2) with a monthly *per capita* income below R$200.00 (OR = 1.5; 95%CI 1.1–2.4). Individuals who reported having harmful habits in the present or past showed higher chances (OR = 17; 95%CI 1.1–2.9) of belonging to a category of greater severity of malocclusion. Among oral health subjective conditions, the following were conditions associated to greater severity of malocclusion: adolescents with negative self-perceived appearance (OR = 1.8; 95%CI 1.1–3.1) and self-perceived social relationship affected by oral health conditions (OR = 1.4; 95%CI 1.1–2.1).

**Table 4:** Results for adjusted analysis of severity of malocclusion among adolescents aged from 15 to 19*. Montes Claros, MG, Southeastern Brazil, 2008-2009.

Variable	Raw analysis			Adjusted analysis		
	
	OR_r_	95%CI	p	OR_a_	95%CI	p
Demographic characteristics and socioeconomic condition						
Self-reported skin color						
Non-white	1.7	1.2–2.4	0.004	1.5	1.1–2.2	0.041
White	1.0	-	-	1.0	-	-
Monthly *per capita* income						
Up to R$200.00	1.7	1.1–2.7	0.028	1.5	1.2–2.4	0.040
Over R$200.00	1.0	-	-	1.0	-	-
Use of dental services and behavior						
Harmful oral habits						
Yes	1.9	1.2–3.1	0.009	1.7	1.1–2.9	0.039
No	1.0	-	-	1.0	-	-
Oral health subjective condition						
Self-perceived appearance of teeth or gums						
Negative	2.2	1.3–3.6	0.003	1.8	1.1–3.1	0.026
Positive	1.0	-	-	1.0	-	-
Self-perceived relationship affected by oral health						
Affects	2.0	1.5–2.6	< 0.001	1.4	1.1–2.1	0.047
Does not affect	1.0	-	-	1.0	-	-

OR_r_: raw odds ratio; OR_a_: adjusted odds ratio

* Deviance test (p = 0.573) and parallel lines test (p = 0.487).

## DISCUSSION

Malocclusion prevalence in the investigated population was 30.4%, and chances of greater severity of malocclusion were higher among socially disadvantaged adolescents who reported having harmful habits and self-perceived compromised aesthetics and social relationship. Regarding the application of health public policy, the epidemiological information presented in this study is useful to adequately prioritize and allocate the necessary resources to provide adolescents with orthodontic treatment.

Despite the methodological rigor of project SB-MOC^14^, the cross-sectional design of this study did not allow an evaluation of causal relations between severity of malocclusion and the factors investigated. A further limitation concerns the sampling process in the rural area, which excluded households outside a 500-meter range from an institution of reference, which may have produced a selection bias, given that 500 m is not a considerable distance in rural areas. This may be the reason for the low percentage (0.4) of adolescents from the rural area in the sample. Therefore, it is prudent to assume that this sample is only representative of the population of adolescents in the urban area of Montes Claros.

We observed a predominance of adolescents with no abnormality or slight malocclusion, followed by those with defined, very severe and severe malocclusion, who require elective, essential and highly necessary orthodontic treatment, respectively. These results are similar to those found in India^16^ and in the 2010 Brazilian epidemiological survey^b^.

Crowding (41.8%) and molar relationship (43.7%) were the most prevalent DAI components. Different results were found in Hungary^10^, where the most prevalent alterations were maxillary irregularity (56.7%), mandibular irregularity (41.8%), and maxillary overjet (60.8%). Approximately 20.0% of adolescents presented maxillary overjet, similar to findings in Lima, Peru^4^, and Recife, in the state of Pernambuco^12^. Most (99.6%) of the adolescents examined did not present mandibular overjet, similar to the findings in Hungary^10^. Expressive prevalence was also observed in spacing in the anterior segment (19.2%) and midline diastema (19.5%), corroborating a previous study. Maxillary and mandibular irregularity affected 22.6% and 24.8% of adolescents, respectively, which are inferior to percentages reported in studies with adolescents in Lima, Peru^4^, and Hungary^10^.

The prevalence of different types of malocclusion observed in this study and in the literature shows wide variability in findings, indicating the need to analyze locally the different treatment requirements of populations. Such variability is possibly related to the multifactorial etiology of malocclusions^8^ or derived from the different measurement instruments adopted to characterize the malocclusions^18^.

The higher probability of severity of malocclusion among adolescents self-reporting as non-white had previously been in reported in Brazil^18^. This association might be caused by the inferior socioeconomic conditions of these ethnic groups compared to the white population of Brazilian society^3^. Youngsters with lower *per capita* income also presented more chances of higher severity of malocclusion when compared to those with higher *per capita* income, an association previously observed in another Brazilian survey^18^. However, Brazilian research evaluating the influence of socioeconomic factors in malocclusion is still scarce.

A complex interrelation among the social factors of oral health seems to exist. Socioeconomic conditions indirectly influence severity of malocclusion by influencing other factors, such as level of education, behavior patterns and access to food, oral hygiene products, and health services, especially orthodontic treatment.

Individuals reporting harmful oral habits showed higher chances of being in a category of greater severity of malocclusion, regardless of demographic characteristics and socioeconomic conditions. Among harmful oral habits, pacifier and thumb sucking may cause malocclusions, since they can alter the normal development of the stomatognathic system, due to an imbalance between external and internal muscle strenght^6^. In this survey, 41.2% of adolescents reported having one or more kinds of harmful oral habits at some moment of their lives, the most reported being nail biting and pacifier or thumb sucking (23.8% and 14.8%, respectively).

In line with the findings of a previous study^5^, adolescents perceiving their appearance as very bad/bad and average showed higher chances of greater severity of malocclusion, as did those perceiving their relationships as affected by oral health conditions. We did not observe any association between severity of malocclusion and self-perceived chewing or speech, suggesting that the malocclusions did not have a perceived functional impact for adolescents. These findings suggest a tendency in individuals to relate malocclusion more closely to aesthetics than functional problems^17^ ^,^ ^20^. It is likely that individuals with malocclusion problems perceived their relationships affected by oral health conditions for being considered less socially attractive, given that, for adolescents, oral aesthetics plays an important role in self-image and social relationships.

Malocclusion was significantly prevalent in the investigated population, and approximately one third of the adolescents showed a need for orthodontic treatment. The malocclusions were associated to variables related to social disadvantage, harmful habits, self-perceived negative appearance, and self-perceived compromised social relationships. Public services in northern Minas Gerais do not offer orthodontic treatment to the population, which evidences problems of access to malocclusion treatment by adolescents whose families are unable to afford the high costs of such treatment in private dental clinics. Consequently, these youngsters may face difficulties of social integration, since malocclusions may represent a social disadvantage for those with no access to treatment. It is essential to improve public policy for the inclusion of orthodontic treatment among health care provided to this segment of the population, particularly among individuals of lower socioeconomic status.

## Footnotes

a Instituto Brasileiro de Geografia e Estatística – IBGE, Cidades@. Minas Gerais: Montes Claros. Rio de Janeiro (RJ): Instituto Brasileiro de Geografia e Estatística, 2010 [cited 2011 Aug 26]. Available from: http://cidades.ibge.gov.br/xtras/perfil.php?lang=&codmun=314330

b Ministério da Saúde. Secretaria de Atenção à Saúde. Secretaria de Vigilância em Saúde. SB Brasil 2010: pesquisa nacional de saúde bucal: resultados principais. Brasília (DF); 2011.
